# Adult T-type lymphoblastic lymphoma presenting as hypercalcemic crisis and aplastic anemia: a case report

**DOI:** 10.1186/s13256-019-2225-2

**Published:** 2019-10-08

**Authors:** Mickael Essouma, Dorothée M. Soh, Mazou N. Temgoua, Ronald M. Gobina, Aristide T. Nono, Etienne Olivier Atenguena, Mahamat Maimouna, Gloria E. Ashuntantang

**Affiliations:** 1 Department of Internal Medicine and Specialties, Faculty of Medicine and Biomedical Sciences, University of Yaoundé I, Yaoundé, Cameroon; 2 Nephrology Unit, Yaoundé General Hospital, Yaoundé, Cameroon; 3 Oncology Unit, Yaoundé General Hospital, Yaoundé, Cameroon

**Keywords:** T-type lymphoblastic lymphoma, Adult, Hypercalcemic crisis, Aplastic anemia

## Abstract

**Background:**

Hypercalcemia and aplastic anemia are two uncommon presentations of non-Hodgkin lymphoma that potentially worsen the disease prognosis. Although hypercalcemia has been reported in the B-cell subtypes and some T-cell subtypes of non-Hodgkin lymphoma, it has not been described in T-cell lymphoblastic lymphoma. The same applies to aplastic anemia, which is also not described in T-type lymphomas.

**Case presentation:**

We report a case of a 52-year-old Cameroonian man with acute kidney injury who presented with confusion, abdominal pain, constipation, polyuria, polydipsia, calciphylaxis, enlarged lymph nodes, tachycardia, and a blood pressure of 170/88 mmHg. Laboratory investigations revealed hypercalcemia (total/ionized 199.5/101.75 mg/L), normal serum phosphorus (40.20 mg/L), and a low intact parathyroid hormone (9.70 pg/ml). Complete blood count revealed pancytopenia. Peripheral blood smear confirmed thrombocytopenia but showed neither blasts nor flower cells. Bone marrow aspirate revealed hypocellularity with no blasts or fibrosis. Lymph node biopsy was suggestive of T-cell precursor lymphoma. T-lymphoblastic lymphoma presenting with hypercalcemic crisis and aplastic anemia was diagnosed, and the patient received the cyclophosphamide-doxorubicin-vincristine-prednisone protocol of chemotherapy together with filgrastim and whole-blood transfusion for aplastic anemia. The short-term outcome was fatal, however.

**Conclusions:**

Severe hypercalcemia and aplastic anemia are potential paraneoplastic syndromes of adult T-type lymphoblastic lymphoma, with fatal short-term outcome.

## Background

T-lymphoblastic lymphoma (T-LBL) is a non-Hodgkin lymphoma (NHL) arising from T-lymphoblasts. It is considered to be rare, because its peak incidence is 3.6 per 100,000 population per year [1]. The typical features of T-LBL that are due to nodal and extranodal infiltration by lymphoblasts are mediastinal mass, peripheral lymph node enlargement, pleural/pericardial effusion, and organ/system (bone marrow, gonads, central nervous system) involvement [2]. Due to wide dissemination at presentation in most patients, the disease prognosis is often poor, with 5-year survival as low as 58.1% during the 2000-2007 period according to a RARECAREnet (Information Network on Rare Cancers) study [3].

Despite extensive reporting of the typical signs of T-LBL in the literature [1], its paraneoplastic syndromes are not well described. Hypercalcemia is a common paraneoplastic syndrome in advanced cases of B-cell subtype NHL [4]. It has also been reported in a few cases of advanced peripheral T-type lymphoma [5–8] and T-cell acute lymphoblastic leukemia/lymphoma (T-ALL) [9, 10]. Although T-ALL and T-LBL share morphological and immunophenotypic similarities [2], no case of hypercalcemia has been reported in T-LBL. Aplastic anemia is another rare disorder associated with bad prognosis in NHL [11] and one that is not described in T-type lymphomas. Accordingly, we report hypercalcemic crisis and aplastic anemia in an adult patient with T-LBL who had a short-term fatal outcome. The purpose of this paper is to create professional awareness of these potentially fatal abnormalities in the course of T-LBL to improve the screening and management of these abnormalities in patients with T-LBL.

## Case presentation

A 52-year-old Cameroonian man, married and a car driver, was admitted to our nephrology inpatient ward for acute kidney injury. He had no relevant past history regarding disease or toxic exposure. He occasionally consumed alcohol and was not a smoker. He had been well until 8 weeks prior to admission, when he developed progressive fatigue, anorexia, involuntary weight loss (~ 12% of his usual body weight), and intermittent nocturnal fever that had been treated twice as malaria and typhoid fever. The malaria and typhoid fever treatments received were parenteral quinine and ofloxacin (doses and duration of treatment not described for both), and both were unsuccessful. A few days before consultation in our hospital, he developed polyuria, polydipsia, constipation, and diffuse abdominal pain that rapidly worsened, prompting consultation at the emergency service of our hospital. The findings of a serum creatinine level of 36.7 mg/L (normal range, < 15 mg/L) and a blood urea nitrogen level of 1.23 g/L (normal range, 0.15–0.45) led to his transfer to the nephrology unit. Upon admission, further questioning revealed that a few days prior to consultation, the patient had inconsistent verbalizations and hallucinations. He was very ill-looking, severely dehydrated, with nontender fixed bilateral inguinal lymph node enlargement (4 cm for the largest), nonreactive urine dipstick, and a fecaloma. His Glasgow Coma Scale score was E4V2M6, blood pressure was 170/88 mmHg, pulse rate was 102/minute, respiratory rate was 17/minute, temperature was 37.7 °C, body surface area was 1.66 m^2^, and random capillary glucose was 0.91 g/L. Apart from the impaired verbal response (in the Glasgow Coma Scale) that limited cognitive function examination, no cranial palsy, meningeal signs, motility abnormalities, sensitivity abnormalities, or coordination abnormalities were found on neurological examination. Cardiac auscultation revealed regular tachycardia. Abdominal examination revealed diffuse tenderness and increased bowel sounds but no defense, contracture, abdominal distention, or visceral enlargement. The result of the rest of the physical examination was normal. Laboratory tests revealed red blood cells 5.4 × 10^12^/L, hemoglobin 14.3 g/dl, white blood cells 6.2 × 10^9^/L, neutrophils 2.6 × 10^9^/L, eosinophils 0, basophils 0, lymphocytes 2.7 × 10^9^/L, monocytes 6.2 × 10^9^/L, platelets 137 × 10^9^/L, total calcium 199.5 mg/L (normal range, 84–105), ionized Ca^2+^ 101.75 mg/L (normal range, 46–54), serum phosphorus 40.20 mg/L (normal range, 25–50), plasma sodium 152 mmol/L (normal range, 135–145), serum potassium 3.1 mmol/L (normal range, 3.5–5), serum chloride 110 mmol/L (normal range, 96–107), and serum magnesium 19.80 mg/L (normal range, 18–26).

**Fig. 1:**
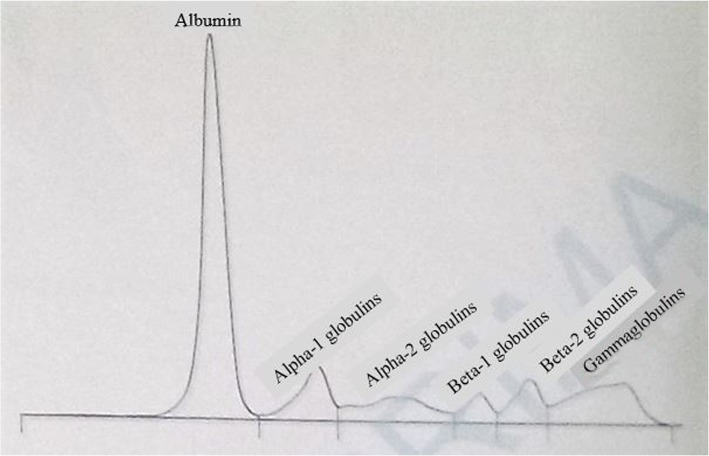
Serum protein electrophoresis curve. Normal total protein and gamma globulin levels, reduced albumin and beta-1 globulins, as well as increased alpha-1 globulins and beta-2 globulins are observed

**Fig. 2:**
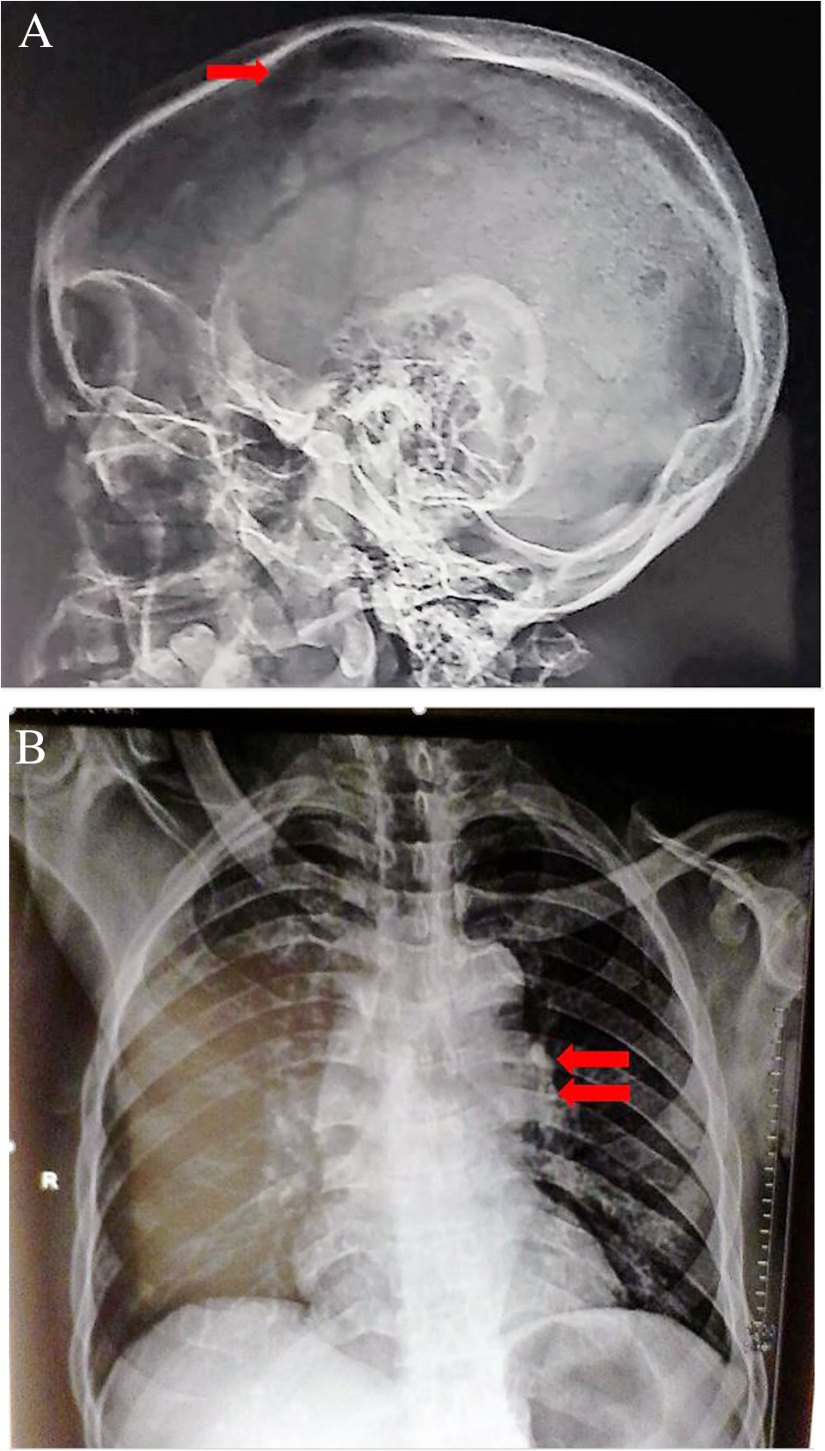
Plain x-rays of the skull and chest radiograph. **a** An osteolytic lesion of the skull (arrow). **b** Bilateral pulmonary hilar lymph nodes (arrows)

**Fig. 3:**
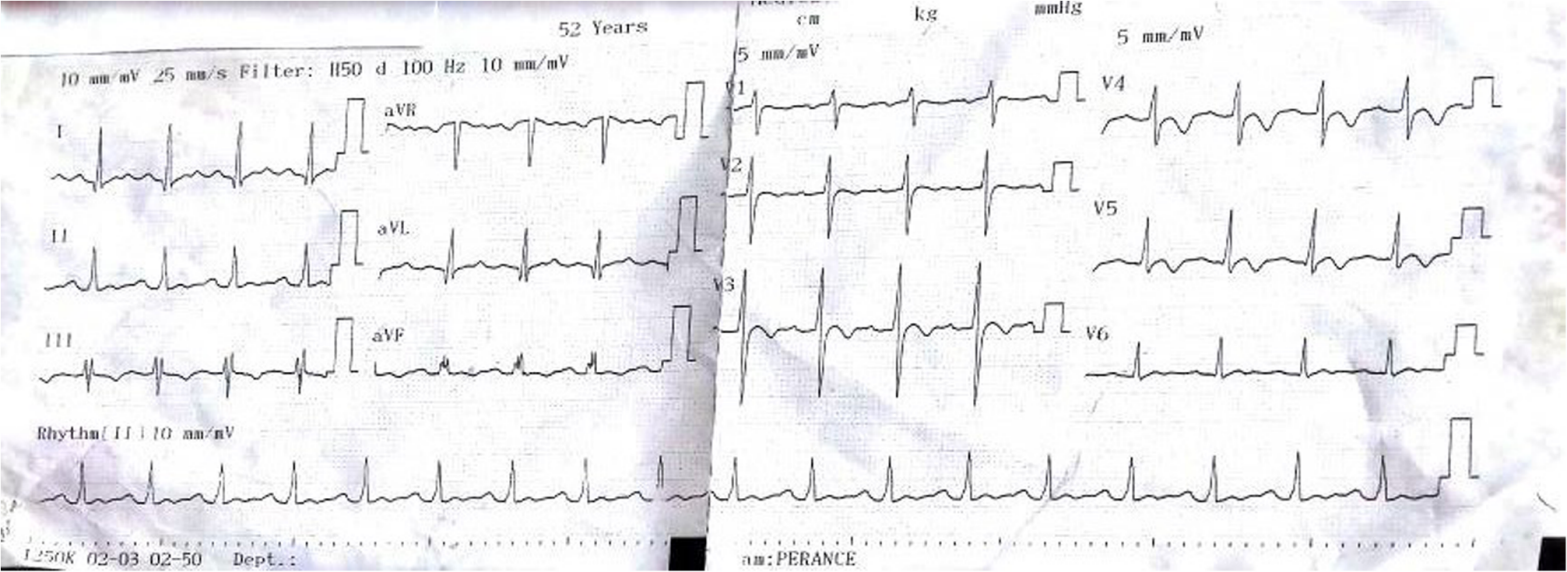
Standard electrocardiogram showing sinus tachycardia at 107 beats per minute

**Fig. 4:**
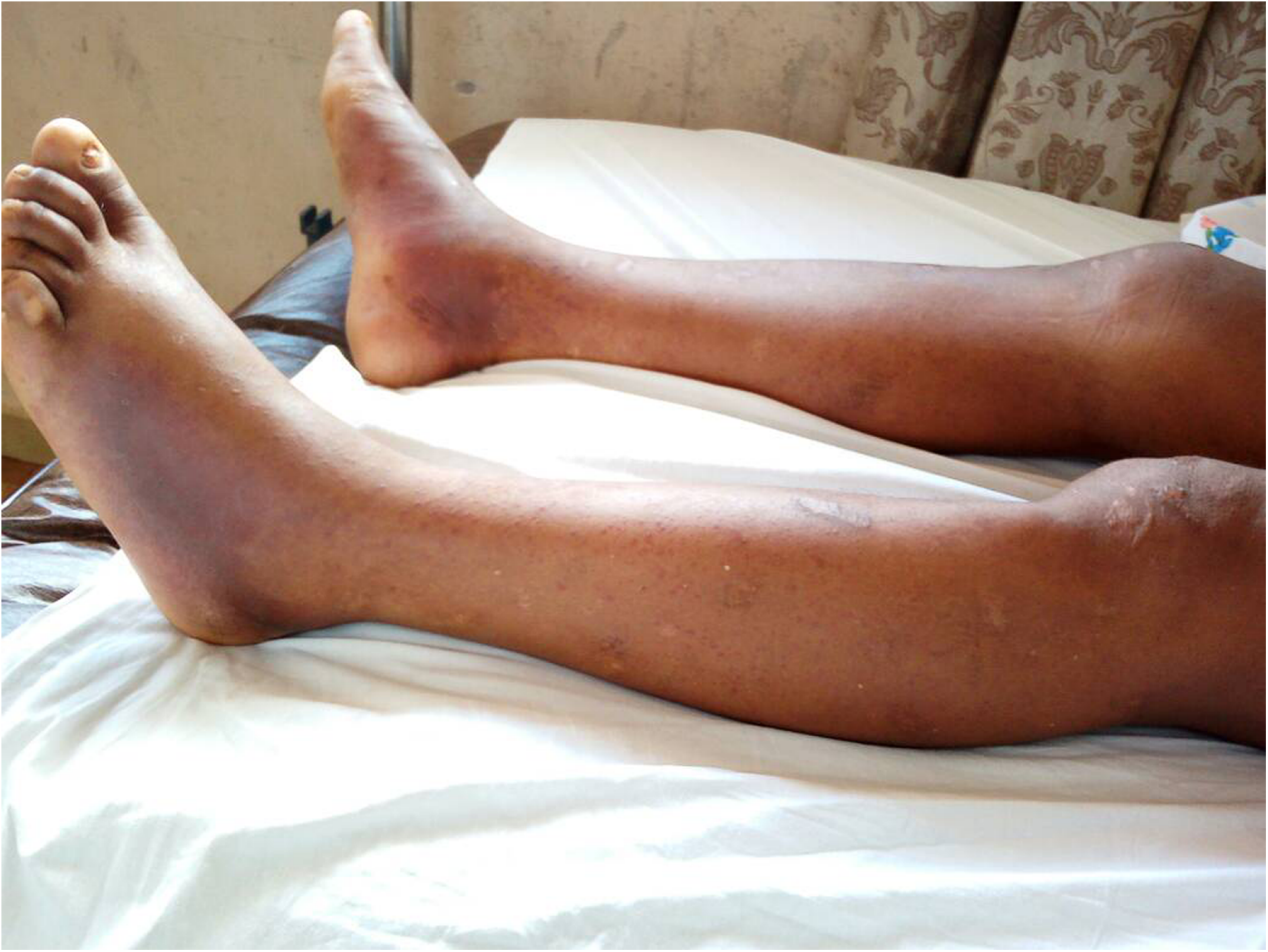
Symmetrical purpura of lower limbs seen during the hypercalcemic crisis, suggestive of nonulcerated peripheral calciphylaxis

**Fig. 5:**
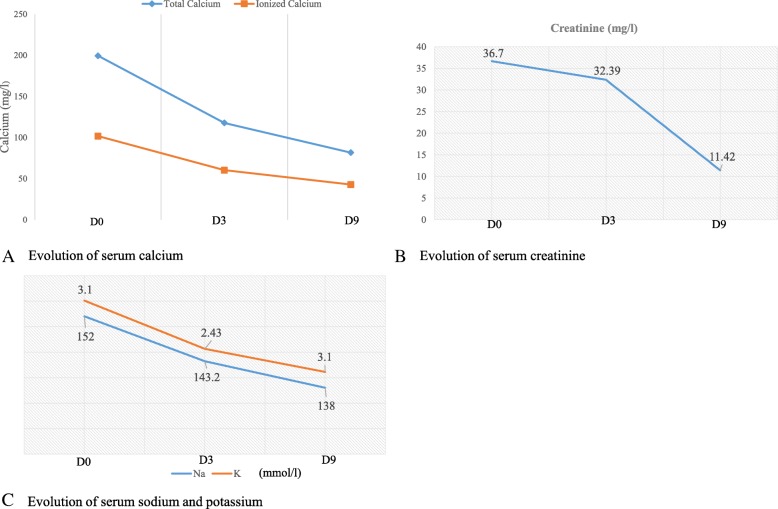
Evolution of serum calcium, creatinine, blood urea nitrogen, sodium, and potassium levels in relation to hypercalcemia treatment. **a** Gradual decrease of serum calcium (total/ionized) from 199.5/101.75 mg/L at beginning of hypercalcemia treatment (D0) to 82/42.84 mg/L 9 days later (D9). **b** Gradual decrease of serum creatinine from 36.7 mg/L at beginning of hypercalcemia treatment (D0) to 11.42 mg/L 9 days later (D9). **c** Gradual decrease of serum sodium and potassium from 152 and 3.1 mmol/L, respectively, at beginning of hypercalcemia treatment (D0) to 138 and 3.1 mmol/L, respectively, 9 days later (D9)

**Fig. 6:**
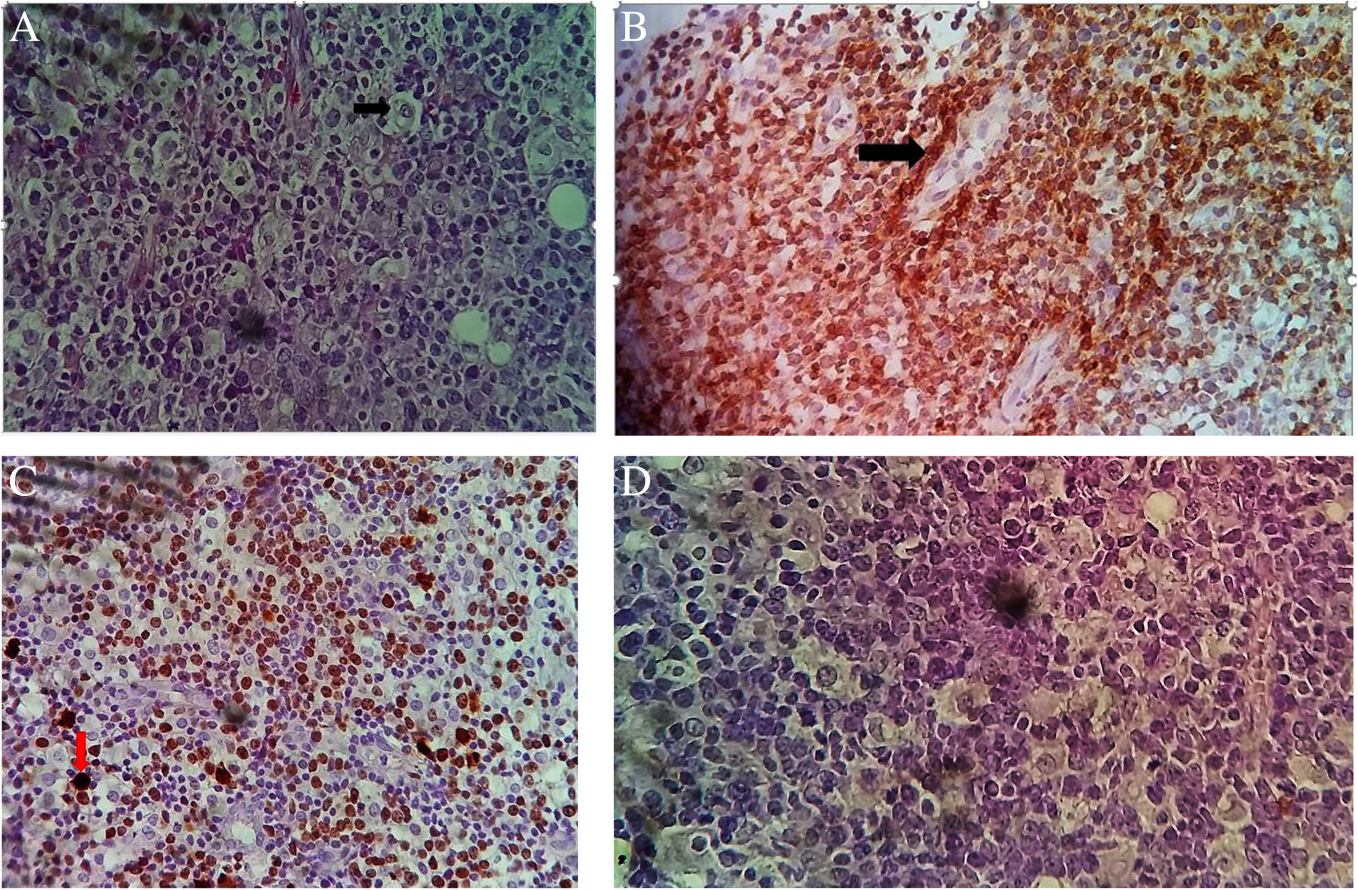
Histological and immunohistochemical features of the excisional lymph node biopsy. **a** Large cells with large nuclei, distinct nucleoli, dispersed chromatin, and scant cytoplasm on hematoxylin and eosin (H&E) staining. **b** Tumor cells positive for cluster of differentiation 3 (CD3) (arrow). **c** Tumor cells positive for Ki67 (arrow). **d** No labeling for cluster of differentiation 20 (CD20)

A provisional diagnosis of a lymphoproliferative disorder or a granulomatous disease causing acute kidney injury was made. Further laboratory tests showed parathyroid hormone (PTH) level 9.70 pg/ml (normal range, 17–73), PTH-related peptide (PTH-rp) level < 8.5 pg/ml (normal range, < 13.0), 1.25(OH)_2_D_3_ level 32 ng/ml (normal range, 18–71 ng/ml for nondialysis subjects), erythrocyte sedimentation rate 50 mm (normal range, < 20 mm), C-reactive protein (CRP) 148.72 mg/L (normal range, < 6 mg/L), and prostate-specific antigen 0.21 ng/ml (normal range, < 4 ng/ml). Figure 1 shows the curve of concurrent serum protein electrophoresis. Plain x-rays of the skull and chest radiographs (Fig. 2a and b) revealed an osteolytic lesion in the skull and mediastinal lymph nodes. A resting electrocardiogram (Fig. 3) confirmed sinus tachycardia as the lone abnormality. Twenty-four hours following admission to the nephrology unit, specific hypercalcemia management was initiated with 4-mg zoledronate once and hydration using intravenous liquids (isotonic saline, 2 L/24 hours and 5% dextrose, 1 L/24 hours) for 3 consecutive days before introduction of oral furosemide 40 mg once daily to correct consequential fluid overload. A symmetric purpura limited to the lower limbs (Fig. 4) progressively developed from the fourth to sixth days of hospitalization, with temperatures reaching a plateau at 40–40.5 °C. On evaluation 9 days after initiation of specific hypercalcemia management, serum calcium and sodium returned to normal values, with complete recovery of related symptoms and signs, as well as renal function (Fig. 5a–c). On the 14th day of hospitalization, although the purpura completely regressed, fever persisted, and lymph node enlargement extended beyond the groins to the cervical region. At this time, results of bacteriological cultures, including urine culture and three serial hemocultures, were all negative, and CRP was 115.61 mg/L. Complete blood count showed red blood cells 2.5 × 10^12^/L, hemoglobin 6.6 g/dl, white blood cells 2 × 10^9^/L, neutrophils 0.6 × 10^9^/L, eosinophils 0.06 × 10^9^/μl, basophils 0, lymphocytes 1.3 × 10^9^/L, monocytes 0.4 × 10^9^/L, and platelets 44 × 10^9^/L. Peripheral blood smear confirmed a low platelet count but showed neither blasts nor flower cells. The reticulocyte count was 22,640/mm^3^. The bone marrow aspirate showed 8.3% normal cellularity with no abnormal infiltrate (including blast cells) or fibrosis. This marrow hypocellularity together with pancytopenia led to the diagnosis of nonsevere aplastic anemia according to the revised Camitta criteria [12]. Inguinal lymph node biopsy showed large cells with distinct nucleoli, dispersed chromatin, and scant cytoplasm with intracytoplasmic CD3 and Ki67 markers, all suggestive of aggressive precursor T-cell lymphoma (Fig. 6a–d). Because lymphoblasts were not observed either in bone marrow analysis or in the peripheral blood smear, and considering histopathologic findings of the lymph node biopsy, T-ALL was ruled out, and the diagnosis of T-LBL was retained [2, 13]. An abdominal ultrasound obtained to assess T-LBL extension showed no organ (spleen, liver, kidney) enlargement. Additional examinations evaluating disease prognosis showed serum lactate dehydrogenase 1145.2 IU/L (normal range, 200–400), uric acid 148 mg/L (normal range, < 70 mg/L), serum glutamic oxaloacetic transaminase 72.3 IU/L (normal range, < 37), and serum glutamic pyruvic transaminase 27.6 IU/L (normal range, < 45). The patient was then transferred to the oncology unit, where he immediately received transfusion of 3 U of whole blood within 2 days prior to chemotherapy. The chemotherapy, which was based on the standard cyclophosphamide-doxorubicin-vincristine-prednisolone (CHOP) protocol for NHL [14], consisted of a 15-minute intravenous infusion of 1250 mg of cyclophosphamide on day 1, a 2-minute intravenous bolus of 80 mg doxorubicin on day 1, a 2-minute intravenous bolus of 2 mg vincristine on day 1, and 100 mg of oral prednisolone once daily on days 1 to 5 of the cycle. Following this CHOP cycle, 1 ml of filgrastim was administered subcutaneously once daily for 3 consecutive days as supportive treatment for aplastic anemia. Resolution of fever and recovery from fatigue and anorexia were noted from the third day of the CHOP cycle. The patient was discharged at the end of the cycle. One week following hospital discharge, he died at home a few hours after fever recurrence.

## Discussion

This paper describes hypercalcemic crisis and aplastic anemia in a middle-aged patient with T-LBL from sub-Saharan Africa who had a short-term fatal outcome. Needless to say, T-LBL is rare and the lowest incidence -0.8 per 100,000 people per year-is recorded in the 25-64 years age group [1]. Hypercalcemic crisis and aplastic anemia were unexpected in this patient, because they have not been described in patients with T-LBL, to the best of our knowledge, unlike lymph node enlargement and bone involvement, which are typical findings in patients with T-LBL [2]. Moreover, T-LBL was revealed by the hypercalcemic crisis.

Severe hypercalcemia and common related signs (confusion, abdominal pain, constipation, nephrogenic diabetes insipidus, acute kidney injury, systolic hypertension, sinus tachycardia) and consequences (hypernatremia and hypokalemia) [15] were notably the most prominent manifestations at presentation. It is remarkable that calciphylaxis, an unusual skin manifestation of hypercalcemia due to peripheral vascular calcification [16], was also observed. Calciphylaxis was evoked due to lower-limb purpura evolving in parallel with serum calcium, but not with platelet counts, and in keeping with the fact that T-LBL-specific treatment had not been started when the purpura had completely regressed [17]. Based on knowledge from other NHLs, hypercalcemia would result from increased 1.25(OH)_2_D_3_ and/or PTH-rp activity [15]. Nonetheless, the normal calcitriol and PTH-rp levels found in this patient could not support that hypothesis. Furthermore, we did not observe an ectopic secretion of PTH. These findings together with the skull osteolytic lesion suggest that hypercalcemia likely resulted from osteolysis.

Aplastic anemia, the second most striking manifestation in this patient, was recently described in 19 patients with B-cell subtypes of NHL in a multicenter European study [11]. The age of our patient corresponds to the median age of patients in that study at the time of lymphoma diagnosis. In addition, seven patients were simultaneously diagnosed with aplastic anemia and lymphoma in that study, as in our patient. Considering all these similarities, we believe that the co-occurrence of both conditions in our patient was not coincidental, even if no specific case of T-lymphoma was reported in the prior study [11]. The putative links between aplastic anemia and lymphoma depend on the presentation sequence of both conditions. Indeed, aplastic anemia is considered an autoimmune paraneoplastic condition when diagnosed simultaneously with lymphoma [11]. However, this is just speculation, and there is a need for in-depth study of the precise mechanistic link.

Beyond the negative impact of some disease-specific factors (age, Ann Arbor stage IVBE, high index of Ki67), aplastic anemia was likely a major poor prognostic factor in this patient. In fact, it has been observed that the outcome of aplastic anemia prevails over that of lymphoma when both conditions co-occur in an individual [11]. Given the probability of stem cell exhaustion and consequential aggravation of aplastic anemia following the CHOP cycle, allogeneic stem cell transplant could have improved the patient’s prognosis [11] if it were available. On the other hand, the conventional CHOP, intensive NHL, and ALL protocols likely provide the same benefit for long-term survival, with less myelosuppression for the conventional CHOP protocol [1]. Besides aplastic anemia, the hypercalcemic crisis was a potential indicator of advanced T-LBL at presentation. Notably, severe hypercalcemia is a classical marker of advanced cancer, and despite the efficacy of calcium-lowering drugs, their likelihood of increasing survival is reduced once hypercalcemia has developed [18]. For instance, the patient described in this paper received the standard of care for hypercalcemia of malignancy, and we observed calcium normalization and improvement of hypercalcemia-related signs before his death. Thus, it is important for the caring physician to accurately anticipate the potential for severe hypercalcemia and aplastic anemia, in view of appropriate therapeutic strategies that can increase the patient’s survival.

There were insufficiencies in the diagnostic and management strategy for this patient due to the lack of resources and adequate infrastructures. Notably, many T-LBL-specific diagnostic and prognostic indicators [19] were not assessed (e.g., CD8, CD25, CD30, and CD56 markers; anaplastic lymphoma kinase; paired box 5; terminal deoxynucleotidyl transferase, and cytogenetic markers). Bone scintigraphy and paraclinical evaluation of the central nervous system (by imaging or lumbar tap) to determine the extent of T-LBL were not carried out. Other causes of aplastic anemia (e.g., folate/cobalamin deficiency) could not be formally ruled out, because they were not investigated. The precise cause of death was not known, because no autopsy was performed. Beyond all these limitations, this paper provides, for the first time, to our knowledge, insights into severe symptomatic hypercalcemia and aplastic anemia with potential short-term negative impact in patients with T-LBL.

## Conclusion

Considering this clinical case, severe hypercalcemia and aplastic anemia may be components of the T-LBL paraneoplastic syndrome. Assessment for those rare presentations early in the course of T-LBL may pave the way to better management and improved survival of affected patients with T-LBL.

## Data Availability

All data supporting our conclusions are included in this report.

## Abbreviations

ALL: Acute lymphoblastic leukemia/lymphoma
CRP: C-reactive protein
NHL: Non-Hodgkin lymphoma
PTH: Parathyroid hormone
PTH-rp: Parathyroid hormone-related peptide
T-LBL: T-lymphoblastic lymphoma
